# Prevalence, Proportions, and Identities of Antibiotic-Resistant Bacteria in the Oral Microflora of Healthy Children

**DOI:** 10.7759/cureus.67277

**Published:** 2024-08-20

**Authors:** Haris Khan, Sher Ahmad Sher, Misbah Iqbal Hanif, Nisar Ahmad Zemawal, Afiyat Ahmad, Farzeen Khan, Muhammad Humayun Daftani

**Affiliations:** 1 Medicine and Surgery, Rehman Medical Institute, Peshawar, PAK; 2 Medicine and Surgery, Lady Reading Hospital, Peshawar, PAK; 3 Pediatric Endocrinology, National Institute of Child Health, Karachi, PAK; 4 Clinical Genetics, Dow University of Health Sciences, Karachi, PAK; 5 Medicine and Surgery, Khyber Medical College, Peshawar, PAK; 6 Community Dentistry, Peshawar Dental College, Peshawar, PAK; 7 Internal Medicine, Lady Reading Hospital, Peshawar, PAK

**Keywords:** klebsiella pneumoniae, enterococcus faecalis, escherichia coli, staphylococcus aureus, oral microflora, children, antibiotic resistant

## Abstract

Background

Antibiotic resistance is a global health concern, yet research on the identities and proportions of antibiotic-resistant bacteria in the oral microflora of healthy children remains limited. These resistant bacteria could play a role in various conditions, such as dental infections, periodontitis, or systemic infections following dental procedures, particularly in immunocompromised individuals. This study aimed to assess the prevalence, proportions, and identities of antibiotic-resistant bacteria in the oral microflora of healthy children.

Methodology

This cross-sectional study, conducted from January to December 2023 across three tertiary care hospitals in Peshawar, Pakistan, involved 364 healthy children aged 7-13 years. Data on antibiotic use were collected via structured interviews and medical records, detailing specific antibiotics (e.g., amoxicillin, ceftriaxone, azithromycin), including dosage, duration, and reason for use. Oral swabs were taken from various sites in the oral cavity using sterile techniques and analyzed using microbiological culture methods and polymerase chain reaction to identify antibiotic-resistant bacteria. Statistical analysis was performed with SPSS version 27.0, utilizing chi-square tests to explore associations between demographic factors and resistance patterns, with significance set at p < 0.05.

Results

Significant proportions of antibiotic use were found among the participants: 172 (47.25%) received penicillins, 91 (25.00%) cephalosporins, and 101 (27.75%) macrolides, with associated side effects including gastrointestinal disorders and allergic responses. Age and gender differences were observed in antibiotic resistance proportions: among 7-9-year-olds, resistance to penicillins was 44 (18.33%), to cephalosporins 29 (12.08%), and to macrolides 33 (13.75%). In contrast, among 10-13-year-olds, these proportions increased to 55 (22.92%), 36 (15.00%), and 43 (17.92%), respectively. Male participants exhibited resistance to cephalosporins at a proportion of 24 (10.00%), to macrolides 45 (18.75%), and to penicillin 46 (19.17%), while female participants showed proportions of 53 (22.08%), 41 (17.08%), and 31 (12.92%), respectively. Among oral swab locations, the buccal mucosa had the highest resistance proportions: 35 (14.58%) to penicillins, 27 (11.25%) to cephalosporins, and 33 (13.92%) to macrolides. Specific bacterial species showed distinct resistance patterns, with notable proportions observed in *Staphylococcus aureus* (n=18; 50.00%, n=12; 33.33%, and n=6; 16.67%, respectively), *Escherichia coli* (n=16; 40.00%, n=10; 25.00%, and n=14; 35.00%, respectively), *Enterococcus faecalis* (n=29; 45.31%, n=14; 21.88%, and n=21; 32.81%, respectively), and *Klebsiella pneumoniae* (n=19; 36.54%, n=13; 25.00%, and n=20; 38.46%, respectively).

Conclusions

This study focused on healthy children aged 7-13 years in Peshawar, Pakistan, to assess the prevalence and types of antibiotic-resistant bacteria in their oral microbiota. The findings highlight resistance patterns by age, gender, and bacterial species. However, the regional context may limit the generalizability of these results. Differences in local antibiotic use, healthcare practices, and environmental factors could influence resistance patterns in other regions. Future research should expand to include diverse geographic locations to evaluate the broader applicability of these findings and identify region-specific factors affecting antibiotic resistance.

## Introduction

The development of antibiotic resistance has become a major worldwide health issue that presents significant obstacles to the efficient treatment of infectious illnesses [1,2]. Although most studies have typically concentrated on antibiotic-resistant bacteria seen in systemic diseases, the oral cavity has a rich ecology that may support the growth of these resistance strains, but it is still mostly unexplored [3]. The oral microbiota of healthy children is a particularly interesting field of research within this niche environment because it acts as a hotspot for microbial interactions and evolution as well as a reservoir for prospective infections [4].

Antibiotic resistance in these children likely develops due to prior exposure to antibiotics, which can lead to the selection and proliferation of resistant bacteria in the oral microbiota. For several reasons, it is essential to comprehend the frequency, sizes, and identities of antibiotic-resistant bacteria in the oral microbiota of healthy children [5]. First, understanding the fundamental makeup of the microbial communities within this population may highlight the processes of bacterial colonization and growth. This information is essential for identifying any changes from typical microbial profiles that may indicate the establishment or spread of pathways for antibiotic resistance [6]. Additionally, as children are more likely than adults to come into contact with and transfer infections in social and educational contexts, research on antibiotic resistance in this population is essential for developing public health initiatives meant to slow the spread of resistant strains [7].

A unique ecological niche is presented by the oral cavity, which is characterized by dynamic microbial interactions affected by environmental exposures, oral hygiene habits, and food [8,9]. As such, examining the frequency of antibiotic-resistant bacteria in this intricate environment requires a multidisciplinary strategy that incorporates epidemiology, clinical research methods, and microbiology [10]. Through the integration of sophisticated molecular approaches with conventional culture-based methodologies, scholars may get a thorough comprehension of the variety and quantity of antibiotic-resistant strains existing in the oral microbiota of well-nourished children [11].

Understanding the distribution of antibiotic-resistant bacteria in healthy children’s oral microflora is crucial for directing evidence-based treatments meant to maintain the effectiveness of currently available antimicrobial medicines, given the rising danger presented by antibiotic resistance [12]. Common oral conditions in children, such as dental infections, abscesses, and severe periodontitis, often require antibiotic treatment, highlighting the need for targeted interventions to mitigate resistance. Healthcare practitioners may reduce the development of antibiotic resistance in communities by implementing targeted treatments after identifying important reservoirs and pathways of transmission of resistant bacteria [13]. To provide the foundation for well-informed measures to counter the growing issue of antibiotic resistance, this research set out to examine the prevalence, quantities, and identities of antibiotic-resistant bacteria within the oral microflora of healthy children.

Research objective

This study aimed to assess the prevalence, proportions, and identities of antibiotic-resistant bacteria in the oral microflora of healthy children and to develop a protocol for managing cases with identified resistant bacteria to guide future clinical practices and antimicrobial stewardship efforts.

## Materials and methods

Study design and settings

This research was conducted across three tertiary care hospitals in Peshawar, Pakistan, namely, Rehman Medical Institute, Lady Reading Hospital, and Hayatabad Medical Complex, serving a diverse population of the Khyber Pakhtunkhwa region. A cross-sectional design was utilized, with the pediatric sections of these hospitals being the primary sites for participant recruitment and data collection. To ensure a comprehensive representation of the target population and account for seasonal variations in antibiotic resistance patterns, the study spanned one year, from January 2023 to December 2023. Efforts were made to include children who may not frequently visit hospitals by conducting outreach programs and collaborating with local clinics to identify eligible participants.

Inclusion and exclusion criteria

The study included healthy children aged 7 to 13 years, with informed consent obtained from their guardians. Children with systemic diseases or medical conditions affecting the oral microbiome were excluded, as were those who had taken antibiotics within the three months before the study. Additionally, participants who were unable or unwilling to provide informed consent were excluded. To analyze seasonal variations, data were categorized by season (winter, spring, summer, fall) and statistical comparisons were made to detect any significant differences in antibiotic resistance patterns across these periods. In addition to the exclusion criteria, participants were assessed for health status through a brief medical history review and a clinical examination by pediatricians to confirm the absence of systemic diseases or conditions affecting the oral microbiome.

Sample size

According to the projected prevalence rates of antibiotic-resistant bacteria in comparable groups, the 364-participant sample size was calculated with a 5% margin of error and a targeted 95% confidence level. The goal of this sample size was to have enough statistical power to identify significant variations in the study population’s patterns of antibiotic resistance.

Data collection

During their hospital stay, guardians of the participants who met the inclusion criteria were contacted, briefed about the study, and informed permission was obtained (appendices). Healthcare personnel with training obtained oral swabs from all participants by using sterile procedures and following normal guidelines. To get a representative sample of the oral microflora, swabs were obtained from many areas of the oral cavity, such as the tongue, gingival crevices, and buccal mucosa. The study included structured interviews and a review of medical records to gather data on antibiotic usage history as well as demographic characteristics such as age, gender, and socioeconomic level. The mode of delivery of antibiotics (oral or parenteral) was recorded to assess its impact on antibiotic resistance patterns.

Laboratory analysis

The oral swabs collected from participants were promptly transported to the microbiology laboratories at the hospitals for thorough examination. Various microbiological culture methods were utilized, including selective media such as MacConkey agar for isolating Gram-negative bacteria and mannitol salt agar for detecting staphylococci. Antibiotic susceptibility testing was conducted using the disk diffusion method. Minimum inhibitory concentrations (MICs) were also determined through broth microdilution techniques to assess the degree of resistance. To further characterize the antibiotic-resistant bacteria, a polymerase chain reaction was employed to identify resistance genes, and DNA sequencing was performed to analyze the genetic profiles and resistance mechanisms. This comprehensive approach ensured a detailed assessment of antibiotic resistance in the oral microbiota of the study participants.

Statistical analysis

The frequency, numbers, and identities of antibiotic-resistant bacteria in the oral microbiota of children in good health were compiled using descriptive statistics in SPSS version 27.0 (IBM Corp., Armonk, NY, USA). The study used chi-square tests to evaluate the correlation between demographic characteristics and patterns of antibiotic resistance. Statistical significance was established at p-values <0.05.

Ethical approval

Rehman Medical Institute, Research Ethics Committee issued study approval (approval number: RMI/RMI-REC/Article Approval/76, September 07, 2022). Guardians of the participants who met the inclusion criteria were contacted and briefed about the study, and informed consent was obtained. The rights, security, and privacy of research participants were guaranteed at all times by adhering to the ethical guidelines specified in the Declaration of Helsinki.

## Results

The frequency of antibiotic classes, widely used antibiotics, patient counts, prevalence percentages, and possible adverse effects are shown in Table 1 for a sample of 364 participants. Amoxicillin and penicillin VK were among the penicillins administered to 47.25% (172 patients) of the participants; both medications have the potential to cause adverse reactions and diarrhea. Of the participants, 25.00% (91 patients) were administered cephalosporins, which included cephalexin and cefuroxime. The possible adverse effects of these antibiotics include allergic reactions and gastrointestinal difficulties. Of the participants, 27.75% (101 patients) were administered macrolides such as azithromycin and clarithromycin, which have the potential to cause allergic responses and gastrointestinal disorders.

**Table 1:** Antibiotic class prevalence in a sample of 364 individuals.

Antibiotic class	Commonly used antibiotics	Number of patients (n)	Prevalence (%)	Potential side effects
Penicillins	Amoxicillin, penicillin VK	172	47.25	Allergic reactions, diarrhea
Cephalosporins	Cephalexin, cefuroxime	91	25.00	Allergic reactions, gastrointestinal disturbances
Macrolides	Azithromycin, clarithromycin	101	27.75	Gastrointestinal disturbances, allergic reactions

**Figure 1:**
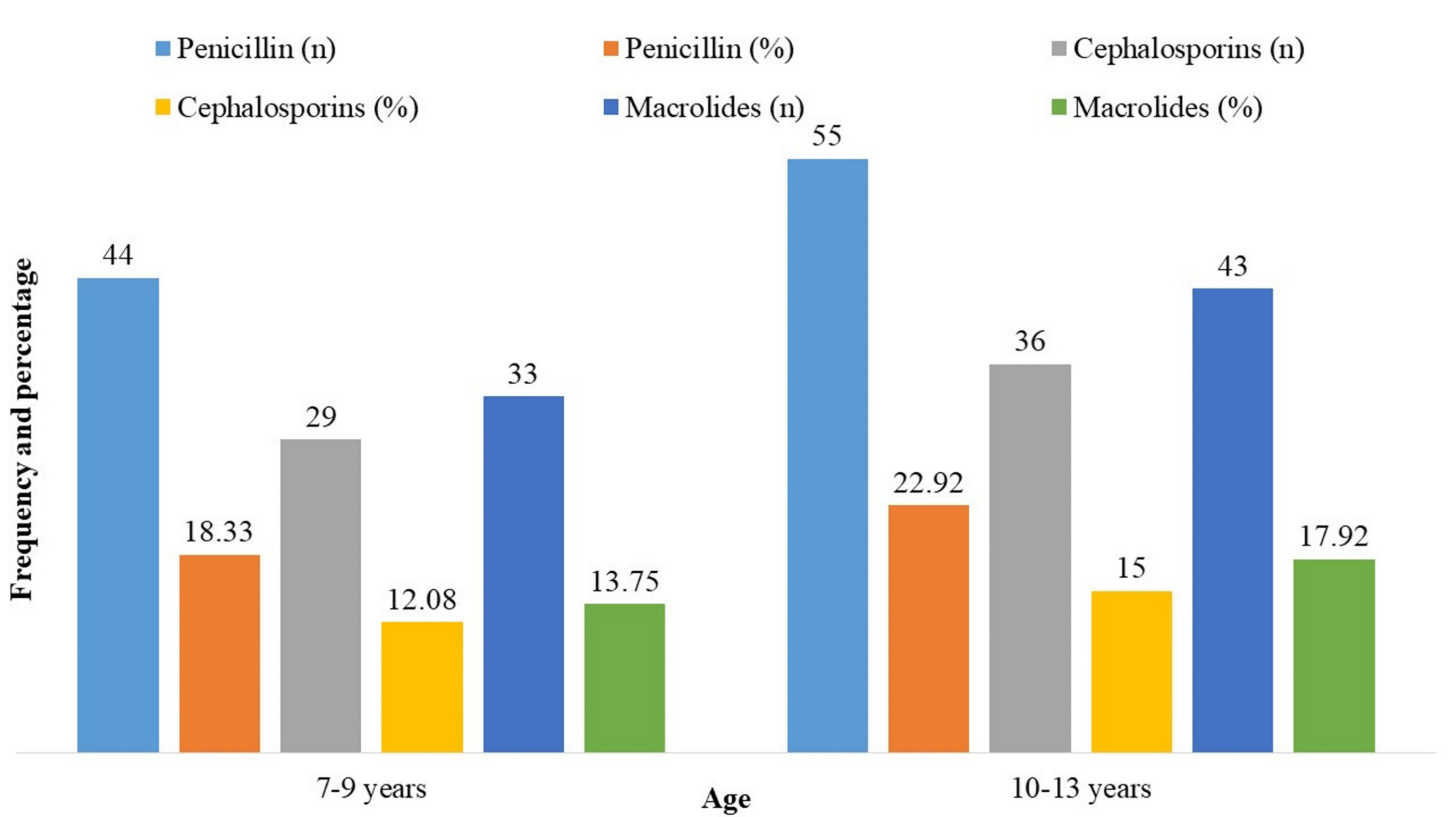
Age group-specific distribution of antibiotic-resistant bacteria (n = 240).

The percentages of microorganisms resistant to antibiotics by age group are shown in Figure 1. Of the 240 patients, 44 (18.33%) were resistant to penicillin, 29 (12.08%) to cephalosporins, and 33 (13.75%) to macrolides in children 7-9 years of age. Of the 240 patients in the 10-13-year age range, 55 (22.92%) had penicillin resistance, 36 (15.00%) cephalosporin resistance, and 43 (17.92%) macrolide resistance. These results point to the need for age-specific antibiotic resistance management techniques by indicating a tendency of rising resistance with age within the examined pediatric group.

**Figure 2:**
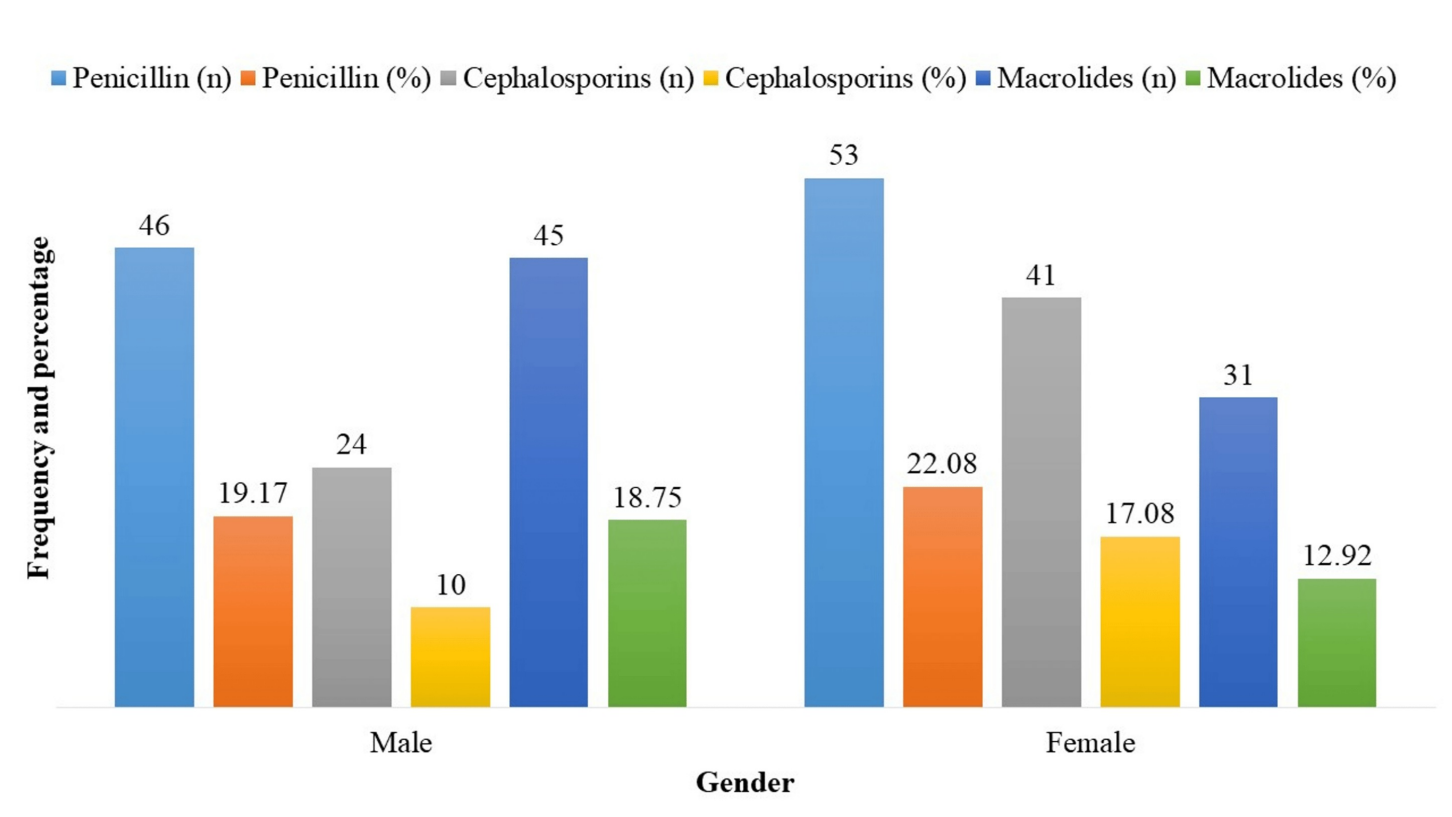
Antibiotic-resistant bacteria prevalence by gender (n = 240).

The frequency of antibiotic-resistant microorganisms broken down by gender is shown in Figure 2. Male patients showed resistance to penicillin (n = 46; 19.17%), cephalosporins (n = 24; 10.00%), and macrolides (n = 45; 18.75%). Female patients demonstrated resistance to penicillin in 53 (22.08%) of cases, cephalosporins in 41 (17.08%), and macrolides in 31 (12.92%) of cases. These gender-based differences emphasize the need for tailored antibiotic resistance interventions.

**Figure 3:**
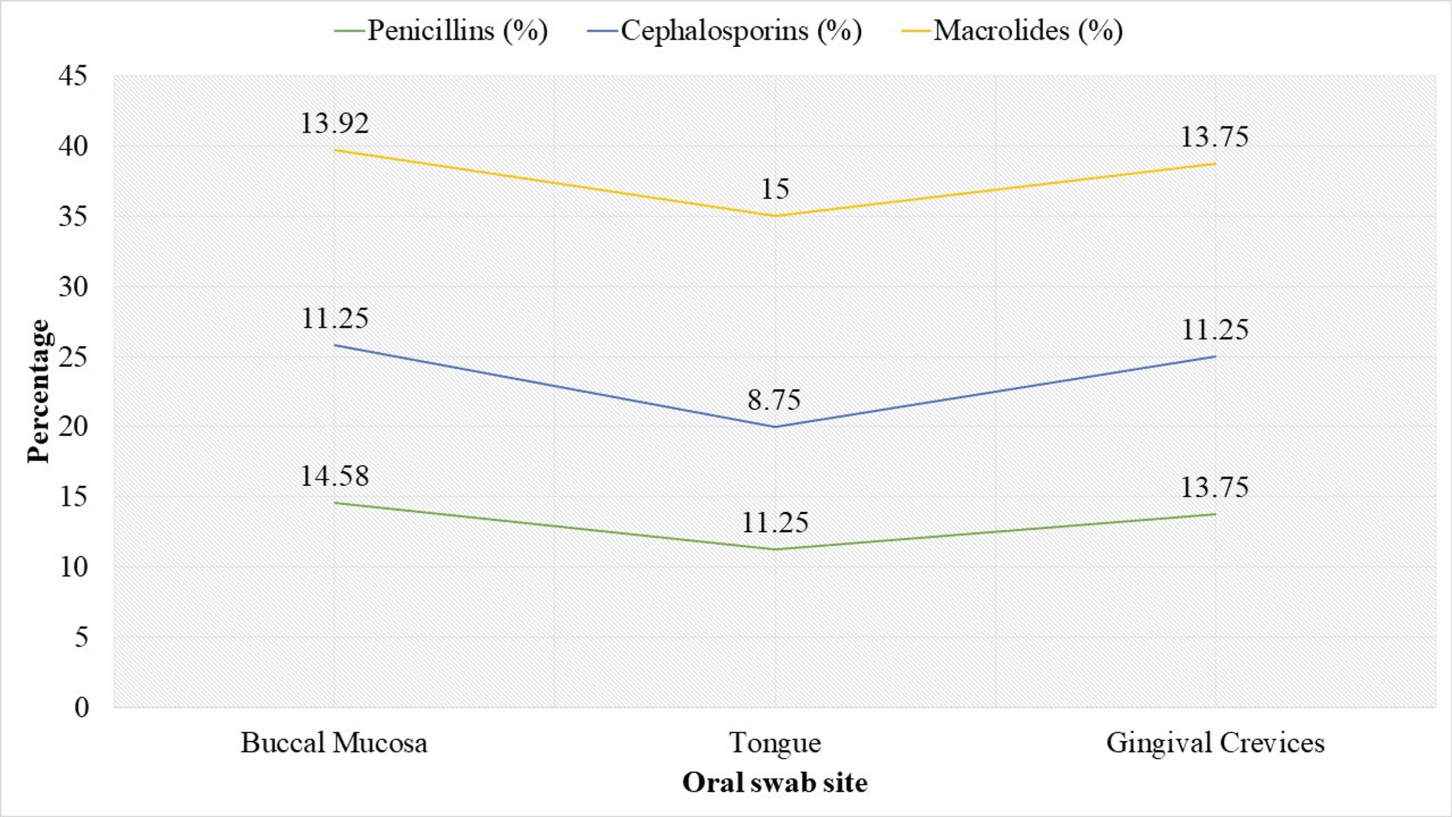
Antibiotic-resistant bacteria prevalence by oral swab sites.

The findings, as shown in Figure 3, revealed differences in the resistance levels between the locations and patient counts. The rates of resistance found in the buccal mucosa were 35 (14.58%), 27 (11.25%), and 33 (13.92%) for penicillins, cephalosporins, and macrolides, respectively. Tongue samples showed somewhat reduced resistance, with rates of 27 (11.25%) for penicillins, 21 (8.75%) for cephalosporins, and 36 (15.00%) for macrolides. Gingival crevices showed resistance rates of 33 (13.75%) for penicillins, 27 (11.25%) for cephalosporins, and 33 (13.75%) for macrolides.

**Figure 4:**
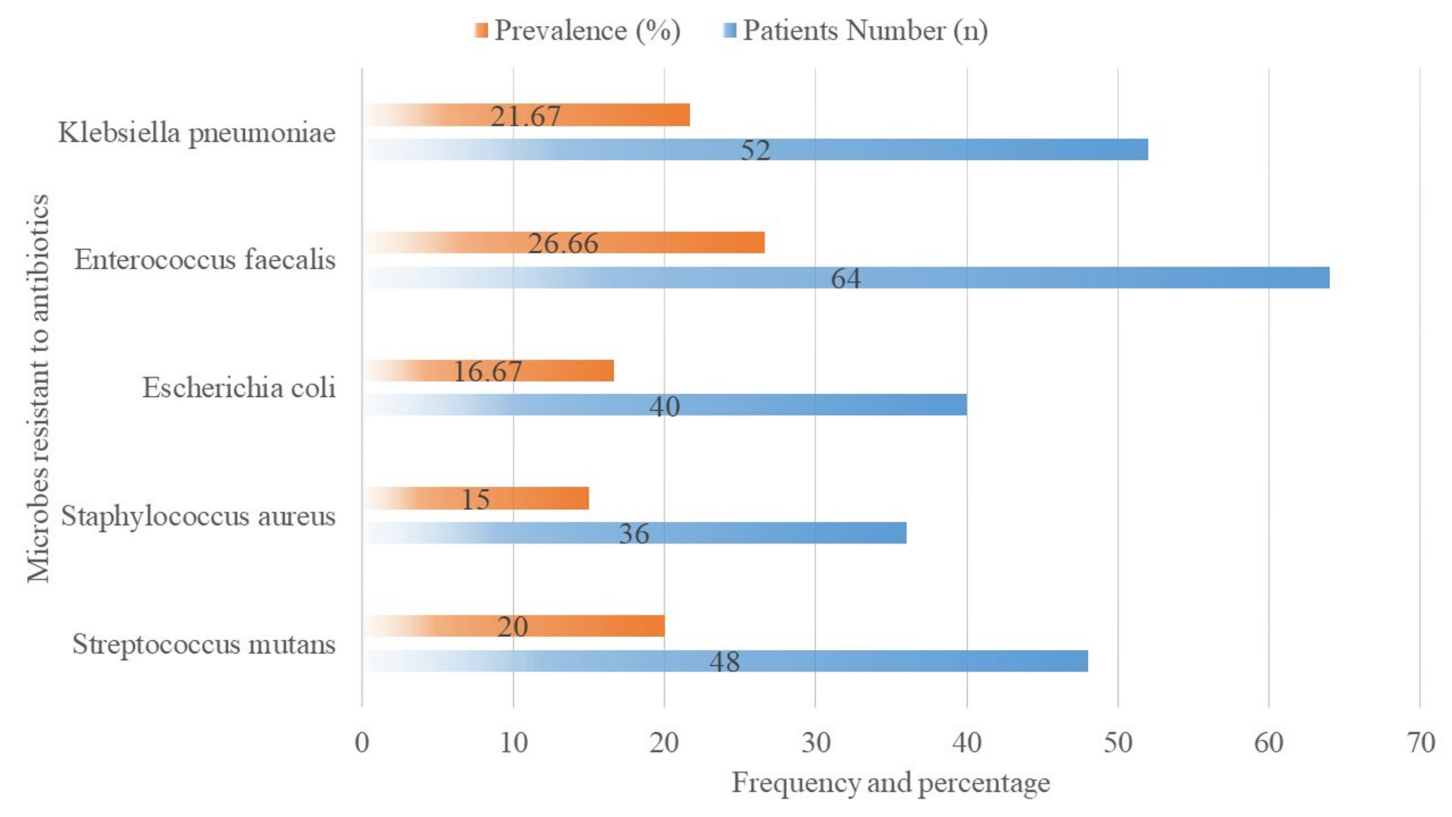
Identifying microbes resistant to antibiotics.

The frequency of bacteria resistant to antibiotics in the oral microbiota of children in good health is shown in Figure 4. Overall, 48 (20.00%) patients had *Streptococcus mutans*, 36 (15.00%) had *Staphylococcus aureus*, 40 (16.67%) had *Escherichia coli*, 64 (26.67%) had *Enterococcus faecalis*, and 52 (21.67%) had *Klebsiella pneumoniae*.

The antibiotic resistance patterns of important bacterial species are shown in Table 2, with percentages and counts for each. With a non-significant p-value of 0.852, 50.00% of *Streptococcus mutants* (n = 48) showed resistance to penicillins, 33.33% to cephalosporins, and 16.67% to macrolides. Similarly, *Staphylococcus aureus* (n = 36) had p-values of 0.859 for resistance rates to penicillins, cephalosporins, and macrolides, respectively, of 50.00%, 33.33%, and 16.67%. With a p-value of 0.618, *Escherichia coli* (n = 40) showed resistance percentages to penicillins, cephalosporins, and macrolides of 40.00%, 25.00%, and 35.00%, respectively. With a p-value of 0.122, which indicates no significant difference, *Enterococcus faecalis* (n = 64) showed resistance rates of 45.31%, 21.88%, and 32.81% to penicillins, cephalosporins, and macrolides, respectively. Finally, with a p-value of 0.480,* Klebsiella pneumoniae* (n = 52) showed resistance percentages to penicillins, cephalosporins, and macrolides of 36.54%, 25.00%, and 38.46%, respectively. This suggests that there is no discernible variation in the resistance patterns among these medications.

**Table 2:** Antibiotic resistance patterns of key bacterial species.

Bacterial species	Penicillin (n; %)	Cephalosporin (n; %)	Macrolides (n; %)	P-value
*Streptococcus mutants* (n = 48)	24 (50.00%)	16 (33.33%)	8 (16.67%)	0.852
*Staphylococcus aureus* (n = 36)	18 (50.00%)	12 (33.33%)	6 (16.67%)	0.859
*Escherichia coli* (n = 40)	16 (40.00%)	10 (25.00%)	14 (35.00%)	0.618
*Enterococcus faecalis* (n = 64)	29 (45.31%)	14 (21.88%)	21 (32.81%)	0.122
*Klebsiella pneumoniae* (n = 52)	19 (36.54%)	13 (25.00%)	20 (38.46%)	0.480

## Discussion

The results of this investigation provide important insights into the numbers, types, and frequency of bacteria resistant to antibiotics in the oral microbiota of children in good health. Penicillins, cephalosporins, and macrolides** **were prescribed, and these findings are consistent with other investigations [5,14], which also noted that penicillins were the most often administered antibiotics in pediatric patients. However, the fact that various antibiotic classes showed varied prescription trends is noteworthy and suggests the need for specialized antimicrobial stewardship approaches. Furthermore, each antibiotic class was associated with possible adverse effects, including allergic responses and gastrointestinal difficulties, highlighting the need to use antibiotics sparingly in young populations.

Different age groups have varying amounts of antibiotic-resistant microorganisms, with a clear pattern of resistance rising with age. The resistance rates rose among children aged 10-13 years. These results underline the significance of age-specific treatments in the fight against antibiotic resistance by indicating age-related changes in antibiotic susceptibility within the pediatric population under study. This is consistent with other research showing that age plays a major role in determining an individual’s sensitivity to antibiotics [15,16].

Moreover, there was a gender difference in the frequency of antibiotic-resistant bacteria, with men showing somewhat lower rates of resistance compared to women. Male patients exhibited resistance to penicillin in 46 cases, cephalosporins in 24 cases, and macrolides in 45 cases. Female patients demonstrated resistance to penicillin in 53 cases, cephalosporins in 41 cases, and macrolides in 31 cases. Previous studies have shown a gender differential in antibiotic resistance patterns, potentially linked to hormonal, immunological, and behavioral differences between males and females [17,18]. Hormonal fluctuations might affect immune responses and lead to variations in resistance patterns, while differing antibiotic usage behaviors may also contribute to these observed differences.

The buccal mucosa showed the highest rates of resistance among the examined locations, with resistance found in 13 cases for macrolides, 11 cases for cephalosporins, and 14 cases for penicillins. The tongue samples had somewhat lower resistance, with 11 cases for penicillins, 8 cases for cephalosporins, and 15 cases for macrolides. Gingival crevices showed resistance rates of 13 cases for penicillins, 11 cases for cephalosporins, and 13 cases for macrolides. These results align with previous research indicating that the buccal mucosa is a key site for microbial colonization and biofilm production [19,20]. The stable environment of the buccal mucosa supports the growth of bacteria, including those that develop and sustain antibiotic resistance. Its complex microenvironment, including interactions with saliva and exposure to oral health products, may contribute to the persistence of resistant strains. Understanding this can help in developing targeted strategies for managing oral microbial resistance.

Patterns of antibiotic resistance in key bacterial species showed varying resistance levels to different antibiotic classes. *Streptococcus mutans* (n = 48) had non-significant p-values of 0.852 with resistance observed in 24 cases for penicillins, 16 cases for cephalosporins, and 8 cases for macrolides. *Staphylococcus aureus* (n = 36) showed similar p-values of 0.859 with resistance in 18 cases for penicillins, 12 cases for cephalosporins, and 6 cases for macrolides. These findings emphasize the need for focused antimicrobial stewardship strategies, including targeted antibiotic use and improved infection control practices, to effectively combat the spread of resistant strains, as highlighted by Helmy et al. [21].

Strengths and limitations

One of the main strengths of this study is its detailed examination of antibiotic-resistant bacteria among a diverse sample of healthy children utilizing a robust cross-sectional design across multiple hospitals. This approach provides valuable insights into age and gender differences in resistance patterns, which are critical for developing targeted interventions. Additionally, the integration of advanced molecular techniques with traditional microbiological methods enhances the reliability of the results.

However, a notable limitation is the exclusion of children who had recently taken antibiotics, which could potentially skew the observed prevalence rates. Despite this limitation, the research significantly advances our understanding of the role of oral microbiota in antibiotic resistance, highlighting the need for age-specific and gender-specific strategies in antimicrobial stewardship. To better understand the applicability of our findings beyond Peshawar, we recommend that future research include diverse geographical areas within Pakistan and internationally. Comparative studies could help determine how the observed phenomena vary across different contexts and identify factors that may affect the generalizability of the results.

## Conclusions

This study on 364 healthy children in Peshawar revealed significant antibiotic resistance in oral bacteria, with notable differences by age, gender, and bacterial species. Resistance rates were higher in children aged 10-13 years compared to those aged 7-9 years, with proportions of 22.92% vs. 18.33% for penicillins, 15.00% vs. 12.08% for cephalosporins, and 17.92% vs. 13.75% for macrolides. Males showed resistance rates of 19.17% for penicillins and 18.75% for macrolides, while females had 12.92% and 17.08%, respectively. The buccal mucosa had the highest resistance proportions: 14.58% to penicillins, 11.25% to cephalosporins, and 13.92% to macrolides. These findings, while critical, are geographically limited to Peshawar and may not be generalizable to other regions due to local variations in antibiotic use and healthcare practices. Future studies should include diverse geographic areas to assess the broader applicability of these results.

## Appendices

**Table 3:** Questionnaire.

Category	Question	Options
Demographic information	What is the child’s age?	
	What is the child’s gender?	Male, Female
	How many family members live in the household?	
Health and medical history	Has the child attended school in the past year?	Yes, No
	Has the child taken any antibiotics in the past six months?	Yes, No
	If yes, please specify the antibiotic(s) taken and the duration of the course.	
	Does the child have any chronic health conditions?	Yes, No
	If yes, please specify the condition(s).	
Hygiene practices	How often does the child brush their teeth?	Twice daily, Once daily, Occasionally, Never
	Does the child use mouthwash regularly?	Yes, No
	Does the child use fluoride toothpaste?	Yes, No
Dietary habits	How often does the child consume sugary foods or drinks?	Frequently, Occasionally, Rarely, Never
Environmental factors	Is there any family member who has been diagnosed with a bacterial infection recently?	Yes, No
	If yes, please specify the bacterial species.	
	Does the child have access to clean drinking water at home?	Yes, No
	Is the child exposed to second-hand smoke?	Yes, No
	Does the child have access to regular dental care?	Yes, No
	What is the socioeconomic status of the family?	Low, Middle, High
Informed consent	I understand the purpose of this study and consent to participate.	Yes, No
	Date:	
	Signature of Parent/Guardian:	
	Name of Parent/Guardian (Optional):	
